# 

**Published:** 1892-02

**Authors:** 


### CONTENTS.

ORIGINAL ARTICLES—Bromide of Ethyl as an Anæsthetic—By E. H. Kuykendall, M. D., Chattanooga, Tenn.................................. 63
The Cure of Epithelioma– By W. S. Gottheil, M. D., New York....... 68
Diseases of Children—By E. Van Goidtsnoven, M. D., Atlanta, Ga.... 73
The Cure of Pulmonary Tuberculosis Upon the Principles of Nutrition —By Karl Von Ruck, M. D., Asheville, N. C.......................... 75
Treatment of a Case of Blood Poison—By S. H. Green, M. D., Oakland, Ga............................................................. 84
CORRESPONDENCE—Our New York Letter.................................. 86
SOCIETY NOTES—The Clinical Society of Maryland...................... 89
EDITORIALS.......................................................... 106–110
Collapse from Perforation of Cæcum or Appendix—Sunday Funerals—Alumni of Southern Medical College—Dr. Harry Cumley—Medical Fortnightly—The Doctors' Weekly.
BOOK REVIEWS........................................................ 93–94
Vest-Pocket Anatomist; By C. Henri Leonard, A. M., M. D—Essentials of Bacteriology; By M. V. Ball, M. D—A Manual of Hypodermatic Medication; By Robert Bartholow, M. D., J. B. Flint & Co.
SELECTIONS AND ABSTRACTS.............................. 85–92—95–105
The Action of Strychnine on the Cerebrum—Fellows Hypophosphites—Peacock's Bromides—Double Pneumonia Complicated with Abortions—An Improved Method of Grafting Ulcers—Treatment of Phosphorus Poisoning—Purulent Vulvo-Vaginitis in Little Girls—Fissures of the Anus in Children—Treatment of Pertussis—Preservation of Meat for Ten Years—For Cold in the Head—A New Method of Tenotomy—Therapie of Huckleberry—Fracture of the Upper Third of Femur—The Value of Bi-Chloride of Mercury in the Treatment of Urethritis—Carpets and Infection—Influenza Colds—Treatment of Ileus—Atropine in Heart Disease.
PRESCRIPTION DEPARTMENT.............................................113–113
Enlarged Spleen—Neuralgia—Dry Bronchial Catarrh—Amenorrhœa—Infantile Diarrhœa—Opium Habit—Iodoform in Acute Gonorrhœa—Irritable Cough—Asthma—Naso-Pharyneal Catarrh—Acute Tonsilitis—Gastric Irritability.
SPECIAL NOTES.......................................................111–112
Sanders & Son—Febriline—Lacto-Preparata—Eucalyptol—Terraline.

